# Long-term safety, tolerability, and efficacy of subcutaneous efgartigimod PH20 in generalized myasthenia gravis: final results from a phase 3 open-label extension study (ADAPT-SC+)

**DOI:** 10.3389/fneur.2026.1876344

**Published:** 2026-08-21

**Authors:** Andreas Meisel, Carlo Antozzi, Ratna Bhavaraju-Sanka, Jan L. De Bleecker, Wan-Yi Huang, Rosa Hermina Jimenez, Fien M. Verhamme, Ming Jiang, Li Liu, Denis Korobko, Anna Kostera-Pruszczyk, Kimiaki Utsugisawa, Jan J. G. M. Verschuuren, Tuan Vu, Heinz Wiendl, James F. Howard

**Affiliations:** 1 Department of Neurology, Charité – Universitätsmedizin Berlin, Berlin, Germany; 2 Neuroimmunology and Neuromuscular Diseases Unit, Fondazione IRCCS Istituto Neurologico Carlo Besta, Milan, Italy; 3 Department of Neurology, University of Texas Health Science Center at San Antonio, San Antonio, TX, United States; 4 Department of Neurology, Ghent University Hospital, Ghent, Belgium; 5 argenx, Ghent, Belgium; 6 State Budgetary Healthcare Institution of Novosibirsk Region “The State Novosibirsk Regional Clinical Hospital”, Novosibirsk, Russia; 7 Department of Neurology, Medical University of Warsaw, Warsaw, Poland; 8 Department of Neurology, Hanamaki General Hospital, Hanamaki, Japan; 9 Department of Neurology, Leiden University Medical Center, Leiden, Netherlands; 10 Department of Neurology, University of South Florida Morsani College of Medicine, Tampa, FL, United States; 11 Department of Neurology, Institute of Translational Neurology, University Hospital Münster, Münster, Germany; 12 Department of Neurology, The University of North Carolina, Chapel Hill, NC, United States

**Keywords:** efgartigimod, FcRn, FcRn antagonist, generalized myasthenia gravis, neonatal Fc receptor, neonatal Fc receptor antagonist

## Abstract

**Background:**

Efgartigimod is a human immunoglobulin (IgG1) antibody Fc fragment that reduces IgG levels through neonatal Fc receptor blockade. ADAPT-SC+ assessed the long-term safety, tolerability, and efficacy of subcutaneous (SC) efgartigimod PH20 in adult participants with generalized myasthenia gravis (gMG). Previously, ADAPT-SC demonstrated noninferiority of efgartigimod PH20 SC to intravenous efgartigimod.

**Methods:**

ADAPT-SC+ was a single-arm, multicenter, open-label extension study. Efgartigimod PH20 SC 1000 mg was administered in treatment cycles of 4 once-weekly injections, with timing of cycles individualized based on clinical evaluation. During the first year of the study, ≥4 weeks were required between cycles; in the second year and onward, participants consenting to a protocol amendment could have ≥1 week between cycles.

**Results:**

A total of 184 participants rolled over from ADAPT+ and ADAPT-SC, and 180 participants received ≥1 dose of efgartigimod PH20 SC. The mean (SD) treatment plus follow-up time was 2.6 (0.9) years, corresponding to 459.4 total participant years of follow-up, with a maximum of 33 treatment cycles. Overall, 169 (93.9%) participants experienced ≥1 treatment-emergent adverse event (TEAE), and the most frequent TEAEs were injection site reactions (ISRs; n = 83, 46.1%), COVID-19 (n = 53, 29.4%), and headache (n = 48, 26.7%). ISRs were mild or moderate in severity. No new safety signals were observed in participants with more frequent dosing (<4 weeks between cycles) after the protocol amendment. Rapid and clinically meaningful improvements (CMI, reduction of ≥2 points) in mean Myasthenia Gravis Activities of Daily Living (MG-ADL) total scores were observed. During the study, 95.1% of participants with acetylcholine receptor antibody–positive (AChR-Ab+) gMG and 94.7% of participants with AChR-Ab− gMG achieved CMI at any point. Many participants demonstrated substantial clinical improvements, with 59.2% of participants with AChR-Ab+ gMG and 34.2% of participants with AChR-Ab− gMG achieving minimal symptom expression (MSE; MG-ADL, 0–1). The majority of participants who achieved MSE (83.3% with AChR-Ab+ gMG, 53.8% with AChR-Ab− gMG) experienced sustained MSE at consecutive assessments covering ≥8 weeks.

**Discussion:**

The results of ADAPT-SC+ demonstrate long-term safety, tolerability, and sustained efficacy of efgartigimod PH20 SC across various dosing approaches, building on previous studies to broaden options for individualizing treatment for patients with gMG.

**Clinical trial registration:**

https://clinicaltrials.gov/study/NCT04818671, NCT04818671.

## 1 Introduction

Generalized myasthenia gravis (gMG) is a rare, chronic autoimmune disease characterized by fluctuating, fatigable weakness affecting multiple muscle groups, which can lead to life-threatening myasthenic crises when exacerbation results in severe respiratory muscle weakness (1, 2). Symptoms result from immunoglobulin G (IgG) autoantibodies, detectable in 85 to 90% of people with gMG, which target components of the neuromuscular junction (3). Most patients with gMG have autoantibodies against the acetylcholine receptor (AChR-Ab+), while about 20% lack anti-AChR autoantibodies (AChR-Ab−). Patients with AChR-Ab− gMG may have autoantibodies against muscle-specific kinase (MuSK) or low-density lipoprotein receptor-related protein 4 (LRP4) or may have no detectable autoantibodies (3). Even in patients who lack detectable autoantibodies, gMG is likely to be autoantibody mediated (4, 5). In patients with AChR-Ab+ gMG, autoantibodies cause disruption of neuromuscular transmission through mechanisms including functional blockade of AChRs, cross-linking, internalization, and degradation of AChRs, and complement activation leading to architectural destruction of the NMJ through membrane attack complex formation (6).

Oral therapies commonly utilized for gMG do not target the molecular mechanisms of gMG pathology but instead consist of symptomatic treatment with acetylcholinesterase inhibitors and broad immunosuppression with corticosteroids and nonsteroidal immunosuppressive therapies (NSISTs) (7). These treatments come with a high burden of adverse side effects and may fail to provide sufficient control of symptoms in many patients (3, 4, 8). Complement protein 5 inhibitors for gMG target only 1 mechanism of gMG pathology and require vaccination against serious meningococcal infections (8, 9). An effective and well-tolerated treatment option that addresses all autoantibody-mediated mechanisms of gMG pathology could address these needs.

Efgartigimod is a human IgG1 antibody Fc fragment with increased affinity for the neonatal Fc receptor (FcRn) relative to endogenous IgG, allowing it to effectively block FcRn-mediated IgG recycling, which results in reduced levels of IgG antibodies, including pathogenic IgG autoantibodies (10–12). Efgartigimod does not affect production of IgG, and patients treated with efgartigimod are able to mount effective immune responses to vaccination (12–15).

Some full-length monoclonal antibody FcRn inhibitors have been shown to disrupt cellular trafficking of FcRn and decrease levels of albumin, which is recycled by FcRn via binding to a site distinct from the IgG binding site (16, 17). Albumin has multiple physiological functions, including roles in cholesterol homeostasis, anticoagulation, fibrinolysis, and vasodilatory ability (17). Decreased albumin levels are associated with increased total and low-density lipoprotein (LDL) cholesterol levels, increased risks of adverse cardiovascular events (including coronary artery disease, myocardial infarction, heart failure, and stroke), and increased risk of kidney function decline and development of chronic kidney disease (18–20). In both the general population and among people with existing cardiovascular risk factors or comorbidities, decreased albumin levels correlate with increased cardiovascular and all-cause mortality risks (19). These effects should be considered when selecting gMG treatment, especially since cardiovascular risk factors and comorbidities may be common in patients with gMG (21, 22). By binding to FcRn in the same location, configuration, and pH-dependent manner as endogenous IgG, efgartigimod does not decrease albumin or increase total and LDL cholesterol levels (12, 16, 17).

The efficacy, safety, and tolerability of intravenous (IV) efgartigimod (10 mg/kg) for treatment of gMG was demonstrated in the pivotal Phase 3 ADAPT trial (NCT03669588), and the open-label extension ADAPT+ (NCT03770403) demonstrated long-term safety and efficacy of efgartigimod IV during up to 17 treatment cycles (10, 11). Subsequently, the subcutaneous (SC) formulation was developed with a higher concentration of efgartigimod coformulated with recombinant human hyaluronidase PH20, which locally degrades hyaluronan to allow rapid (30–90 s) SC administration of the 5.6-mL injection volume (23, 24). In the Phase 3 randomized, open-label ADAPT-SC study (NCT04735432), efgartigimod PH20 SC 1000 mg demonstrated noninferiority to efgartigimod IV 10 mg/kg in percent reduction of total IgG at Week 4, 1 week after the last injection of the first treatment cycle, and the formulations had comparable efficacy and safety profiles (23). Interim results from the single-arm, open-label extension ADAPT-SC+ (NCT04818671) demonstrated safety and efficacy of efgartigimod PH20 SC treatment during up to 6 treatment cycles (23). Efgartigimod (IV and/or PH20 SC) is approved for treatment of gMG and chronic inflammatory demyelinating polyradiculoneuropathy (CIDP) in multiple countries and regions and for treatment of primary immune thrombocytopenia (ITP) in Japan (25–28).

Here, we present results from the final analysis of the ADAPT-SC+ study to evaluate the long-term safety, tolerability, and efficacy of efgartigimod PH20 SC in participants with gMG during up to 3.7 years of treatment. This includes participants who received more frequent dosing of efgartigimod relative to previous gMG studies, with individualized dosing allowing as few as 7 days between treatment cycles after the first year of the study.

## 2 Materials and methods

### 2.1 Study design

**Figure 1:**
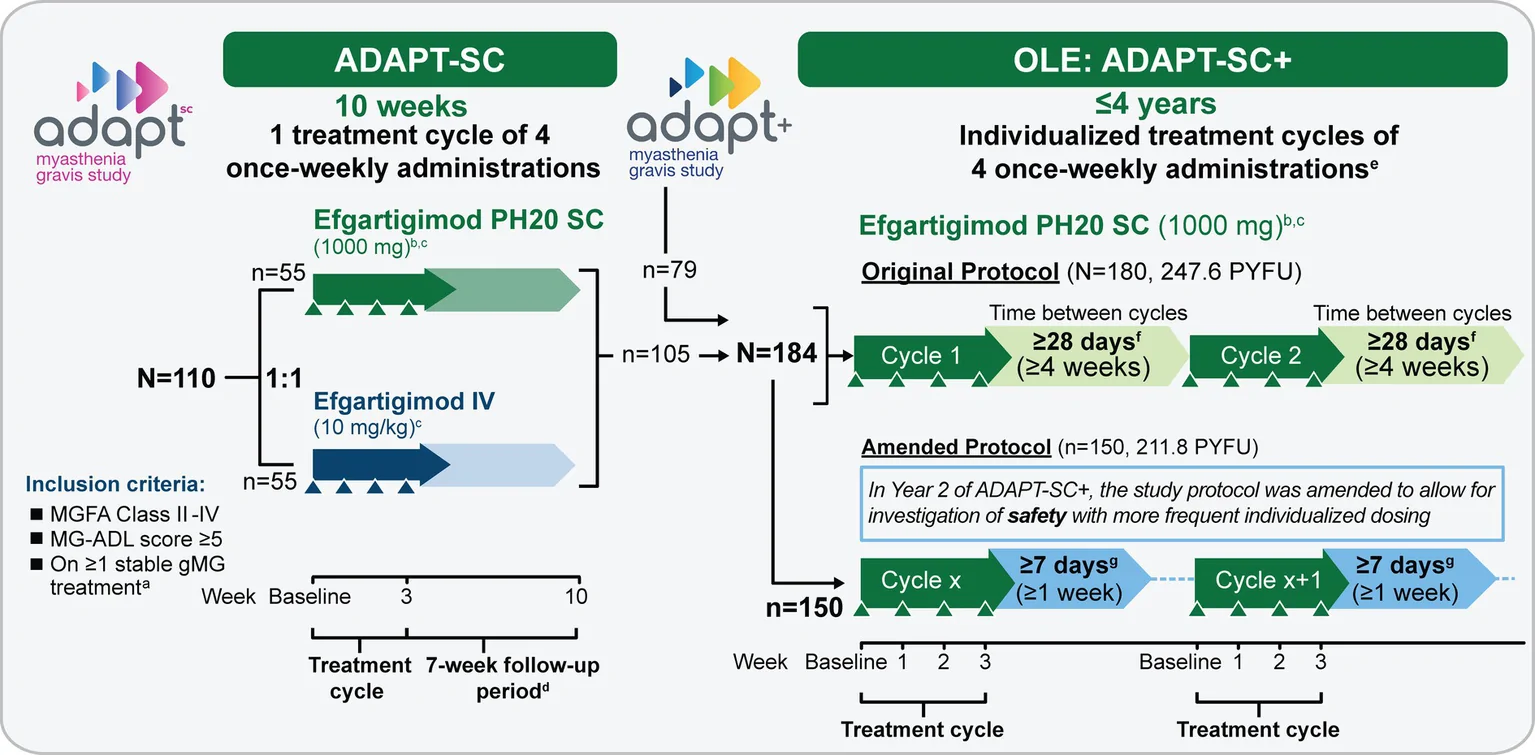
Study design: AChEI, acetylcholinesterase inhibitor; gMG, generalized myasthenia gravis; IV, intravenous; MG-ADL, Myasthenia Gravis Activities of Daily Living; MGFA, Myasthenia Gravis Foundation of America; NSIST, nonsteroidal immunosuppressive therapy; PYFU, participant years of follow-up; rHuPH20, recombinant human hyaluronidase PH20; SC, subcutaneous. ^a^Stable concomitant therapies included NSISTs initiated ≥6 months prior to screening with stable dose for ≥3 months prior to screening, corticosteroids initiated ≥3 months prior to screening with stable dose for ≥1 month prior to screening, and AChEIs initiated ≥2 weeks prior to screening with no dose escalation in the 2 weeks prior to screening. ^b^Coformulated with 2000 U/mL rHuPH20. ^c^Green triangles indicate efgartigimod PH20 SC injection, and blue triangles indicate efgartigimod IV infusion. ^d^Participants did not receive efgartigimod during the 7-week follow-up period. ^e^Participants not in need of retreatment at study entry instead started with an intertreatment period. ^f^At least 28 days between the last dose of the previous treatment cycle and the first dose of the next treatment cycle, based on the need for treatment as determined by the investigator. ^g^In participants who consented to the protocol amendment, treatment cycles during and after Year 2 could be administered with ≥7 days between the last dose of the previous treatment cycle and the first dose of the next treatment cycle, at the discretion of the investigator based on clinical evaluation.

ADAPT-SC+ was a single-arm, multicenter, open-label extension of the Phase 3 ADAPT-SC study conducted between April 26, 2021, and December 31, 2024. Participants were enrolled at 47 study sites worldwide. Efgartigimod PH20 SC 1000 mg was administered in treatment cycles of 4 once-weekly injections, and the number of treatment cycles and time between cycles (also referred to as an intertreatment period) was individualized based on clinical evaluation. Participants were not required to have clinical worsening of symptoms to be eligible for subsequent cycles. Under the original study protocol, ≥28 days were required between the last injection of a treatment cycle and the first injection of the subsequent cycle. To allow further individualization of dosing and management of fluctuations in disease severity that may occur <4 weeks after the final injection of a treatment cycle, a protocol amendment was implemented on February 2, 2023, which allowed shorter intervals (≥7 days) between cycles during the second year and onward at the investigator’s discretion (Figure 1).

ADAPT-SC+ was conducted in accordance with the Declaration of Helsinki and Council for International Organizations of Medical Sciences international ethical guidelines, applicable International Council for Harmonization Good Clinical Practice guidelines, and other applicable local laws and regulations. The original and amended study protocols were approved by institutional review boards and/or independent ethics committees prior to study initiation and prior to the protocol amendment becoming available. All participants provided written informed consent prior to entering the study and prior to treatment under the amended study protocol. Study data are presented in accordance with STROBE guidelines for cohort studies (29).

### 2.2 Participants

Participants who previously participated in either the ADAPT-SC or ADAPT+ study were eligible to enroll in ADAPT-SC+. As in the antecedent studies, participants were required to be on a stable dose of ≥1 concomitant therapy for gMG. Permitted concomitant therapies included NSISTs (stable dose for ≥3 months prior to retreatment with efgartigimod PH20 SC in ADAPT-SC+), corticosteroids (stable dose for ≥1 month prior to retreatment), and acetylcholinesterase inhibitors (stable dose for ≥2 weeks prior to retreatment). Prohibited therapies for participants in ADAPT-SC+ included monoclonal antibodies, experimental agents other than efgartigimod PH20 SC, and live/live-attenuated vaccines. Full inclusion and exclusion criteria for study participation are listed in Supplementary Table 1.

### 2.3 Outcomes

The primary objective of ADAPT-SC+ was to investigate the long-term safety and tolerability of efgartigimod PH20 SC in participants with gMG. Endpoints to evaluate safety and tolerability included incidence and severity of treatment-emergent adverse events (TEAEs), incidence of severe TEAEs, and changes in laboratory test results, physical examination results, vital signs, and electrocardiogram readings.

The impact of efgartigimod PH20 SC on disease severity was measured by observing changes in Myasthenia Gravis Activities of Daily Living (MG-ADL) total scores throughout the study. The percentages of participants experiencing clinically meaningful improvement (CMI) and minimal symptom expression (MSE) were calculated based on MG-ADL total scores: a CMI in MG-ADL total score was defined as a reduction of ≥2 points, and MSE was defined as an MG-ADL total score ≤1. Improvements in quality of life (QoL) among participants were assessed using Myasthenia Gravis Quality of Life 15-Item Questionnaire, Revised (MG-QoL15r) scores during the first year of the study.

The pharmacodynamic effect of efgartigimod PH20 SC was assessed by measuring levels of total IgG in all participants and AChR-Ab in AChR-Ab+ participants during the first year of the study (up to 12 treatment cycles).

### 2.4 Statistical analysis

Analyses included data from all participants who received ≥1 dose of efgartigimod PH20 SC during the study. TEAEs were summarized by Medical Dictionary for Regulatory Activities system organ class and preferred term, by severity, by participant outcome, and by relationship to efgartigimod PH20 SC treatment. These summaries were reported for the participant population overall, by AChR-Ab serostatus, and for the population of participants who consented to the protocol amendment (before and after the protocol amendment and by average duration of time between cycles after the protocol amendment). The Common Terminology Criteria for Adverse Events (criteria version 5.0) was used to grade the severity of adverse events. Clinical laboratory evaluations were summarized using descriptive statistics, which were calculated for the participant population overall. Efficacy and QoL data were summarized as changes in scores from baseline over time using descriptive statistics for the overall population by AChR-Ab serostatus. Pharmacodynamic data were summarized for each cycle using descriptive statistics for the overall population by AChR-Ab serostatus. Across all analyses, missing data were not imputed.

## 3 Results

### 3.1 Participants

**Figure 2:**
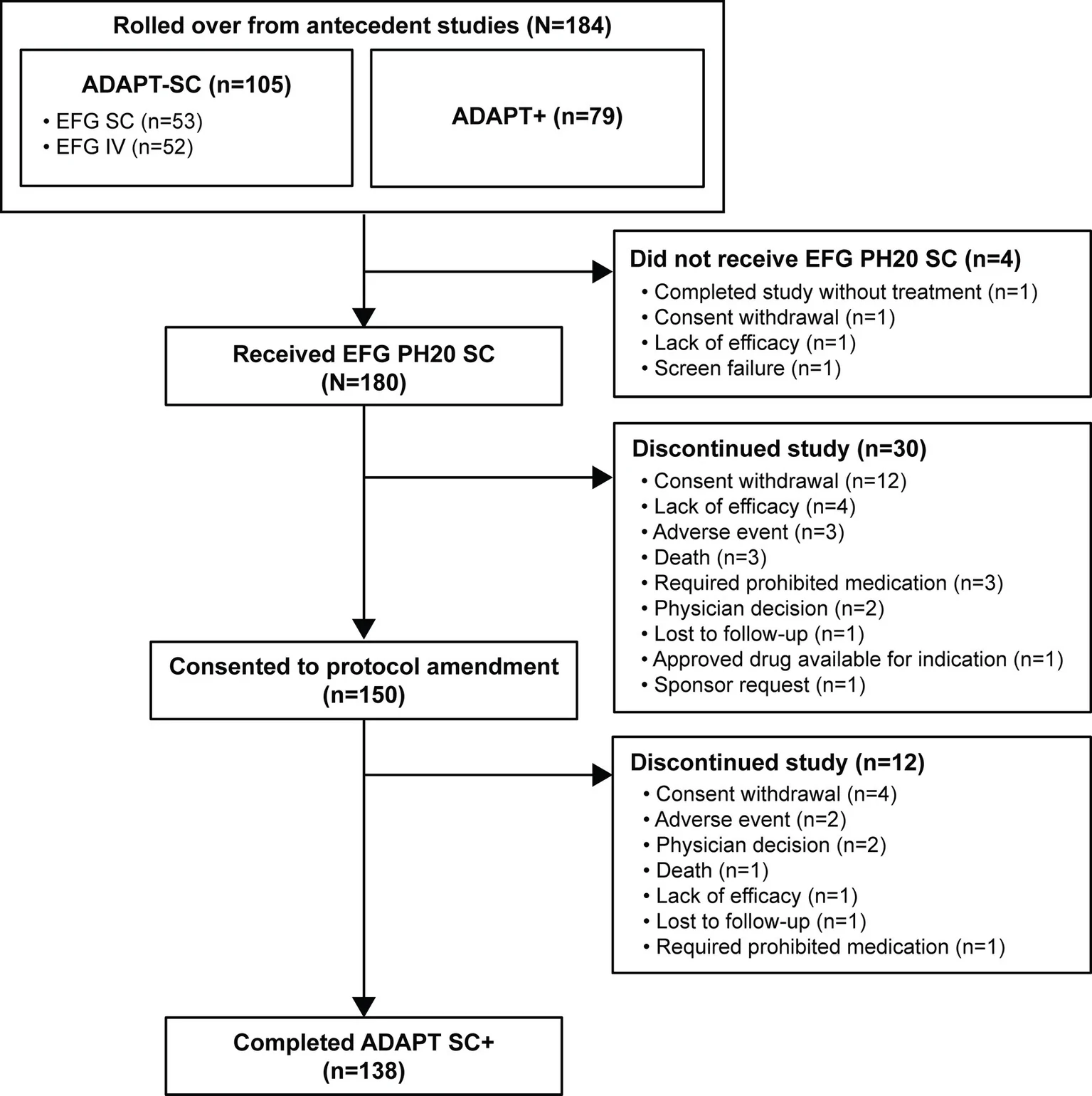
Participant disposition. EFG, efgartigimod; IV, intravenous; SC, subcutaneous.

Of 184 participants who rolled over from antecedent studies (*n* = 105 from ADAPT-SC, *n* = 79 from ADAPT+), 180 participants received ≥1 dose of efgartigimod PH20 SC during ADAPT-SC+ and were included in the safety and efficacy analyses (Figure 2). Of participants who received efgartigimod, 150 (83.3%) provided informed consent for the protocol amendment allowing less time between treatment cycles. During ADAPT-SC+, 42 participants discontinued the study; the most common reasons for discontinuation were withdrawal of consent (*n* = 16, 8.9%), adverse events (*n* = 5, 2.8%), and lack of efficacy (*n* = 5, 2.8%). Other reasons for discontinuation are reported in Figure 2.

Participant demographics and baseline disease characteristics are reported in Table 1. Most participants (*n* = 142, 78.9%) were AChR-Ab+, and the remaining participants (*n* = 38, 21.1%) were AChR-Ab−. The demographics and baseline characteristics of the participant population that consented to the amended protocol allowing less time between treatment cycles were similar to the overall participant population (Supplementary Table 2).

**Table 1:** Demographics and baseline characteristics.

Characteristic	Overall population (*N* = 180)	AChR-Ab+ population (*n* = 142)	AChR-Ab− population (*n* = 38)
Age (y)			
Mean (SD)	50.8 (15.5)	51.1 (15.9)	49.7 (14.2)
Median (min, max)	50.0 (19, 84)	52.5 (19, 84)	49.5 (22, 79)
Sex, *n* (%)			
Female	119 (66.1)	90 (63.4)	29 (76.3)
Male	61 (33.9)	52 (36.6)	9 (23.7)
Race, *n* (%)			
Asian (Japanese)	16 (8.9)	11 (7.7)	5 (13.2)
Black or African American	2 (1.1)	1 (0.7)	1 (2.6)
Multiple	1 (0.6)	1 (0.7)	0 (0.0)
White	161 (89.4)	129 (90.8)	32 (84.2)
Weight (kg)			
Mean (SD)	78.9 (20.2)	79.2 (20.7)	77.5 (18.7)
Median (min, max)	77.0 (42.0, 148.8)	77.0 (42.0, 148.8)	76.1 (45.9, 146.6)
Time since gMG diagnosis (y)			
Mean (SD)	9.0 (8.0)	9.1 (8.5)	8.6 (5.7)
Median (min, max)	6.0 (0.4, 48.1)	5.9 (0.4, 48.1)	7.2 (0.7, 20.3)
Previous thymectomy, *n* (%)			
Yes	68 (37.8)	58 (40.8)	10 (26.3)
MGFA class at screening, *n* (%)			
Class II	73 (40.6)	59 (41.5)	14 (36.8)
Class III	100 (55.6)	78 (54.9)	22 (57.9)
Class IV	7 (3.9)	5 (3.5)	2 (5.3)
MG-ADL total score at baseline			
Mean (SD)	7.8 (3.4)	7.5 (3.4)	8.9 (3.4)
Median (min, max)	8.0 (0, 20)	7.0 (0, 20)	8.5 (0, 15)
MG-QoL15r total score at baseline			
Mean (SD)	13.6 (6.9)	13.1 (6.8)	15.5 (6.8)
Median (min, max)	13.0 (0, 30)	12.5 (0, 30)	14.5 (0, 28)
AChR-Ab status, *n* (%)			
AChR-Ab+	142 (78.9)	142 (100.0)	0 (0.0)
AChR-Ab−	38 (21.1)	0 (0.0)	38 (100.0)
Concomitant^a^ MG therapy, *n* (%)			
Any steroid	128 (71.1)	103 (72.5)	25 (65.8)
Any NSIST	90 (50.0)	68 (47.9)	22 (57.9)
Any AChEI	150 (83.3)	122 (85.9)	28 (73.7)
Steroid + NSIST	69 (38.3)	53 (37.3)	16 (42.1)
AChEI only	29 (16.1)	23 (16.2)	6 (15.8)

^a^During the first year of ADAPT-SC+. AChEI, acetylcholinesterase inhibitor; AChR-Ab, acetylcholine receptor antibody; gMG, generalized myasthenia gravis; MG, myasthenia gravis; MG-ADL, Myasthenia Gravis Activities of Daily Living; MGFA, Myasthenia Gravis Foundation of America; MG-QoL15r, Myasthenia Gravis Quality of Life 15-Item Questionnaire, Revised; NSIST, nonsteroidal immunosuppressive therapy.

### 3.2 Safety

The mean (SD; minimum, maximum) treatment plus follow-up time among participants in ADAPT-SC+ was 2.6 (0.9; 0.2, 3.7) years, leading to a total [suggested edit] of 459.4 participant years of follow-up (PYFU). The maximum number of treatment cycles was 33 (*n* = 8 participants). During the study, the mean (SD) total number of injections per participant was 64.7 (37.4), corresponding to approximately 16 treatment cycles per participant.

**Figure 3:**
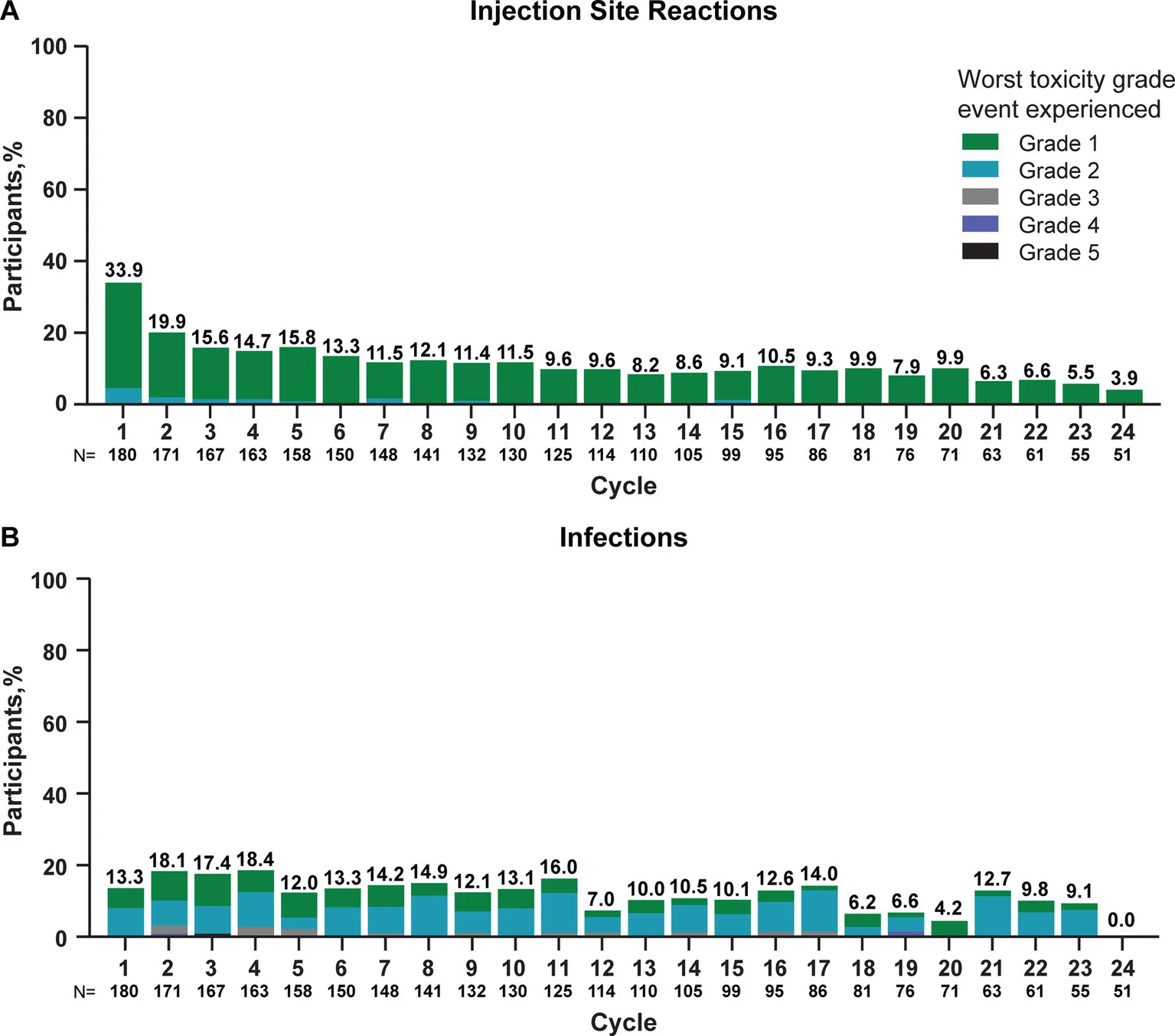
Treatment-emergent adverse events by treatment cycle. **(A)** Percentage of participants experiencing injection site reactions in each cycle. **(B)** Percentage of participants experiencing infections in each treatment cycle.

Over the course of the study, 169 (93.9%) participants experienced ≥1 TEAE (Table 2). The most frequently reported TEAEs were injection site reactions (ISRs; *n* = 83, 46.1%), COVID-19 (*n* = 53, 29.4%), and headache (*n* = 48, 26.7%). All headaches were mild or moderate in severity, and none led to treatment discontinuation. Serious TEAEs were experienced by 55 (30.6%) participants (Table 2), and the most common serious TEAE was MG worsening (*n* = 11, 6.1%). Five participants (2.8%) died during the ADAPT-SC + study period, and the fatal TEAEs (metastatic renal cell cancer, cardiac arrest, respiratory failure [*n* = 2], and COVID-19/respiratory failure) were considered not related to efgartigimod PH20 SC treatment by the investigators. TEAEs leading to treatment discontinuation occurred in 8 (4.4%) participants, and none of these events were considered to be related to efgartigimod PH20 SC treatment by the investigators. ISRs were all mild or moderate (grade 1 or 2) in severity. The majority of ISRs observed were erythema (57.6%); injection site edema (9.0%) and injection site swelling (7.9%) were the next most frequently reported. The percentage of participants experiencing ISRs in each cycle decreased over subsequent treatment cycles (Figure 3A). Infections were experienced by 121 (67.2%) participants, and the most frequently reported infections were COVID-19 (*n* = 53, 29.4%), nasopharyngitis (*n* = 36, 20.0%), and upper respiratory tract infection (*n* = 32, 17.8%) (Table 2). Most infections were mild or moderate in severity, and incidence of infections did not increase with additional treatment cycles (Figure 3B).

**Table 2:** Safety.

Event type	Overall population (*N* = 180, 459.4 PYFU)	
	*n* (%)	ER^a^
Any TEAE	169 (93.9)	7.2
Any TEAE grade ≥3	62 (34.4)	0.3
Any serious TEAE	55 (30.6)	0.2
Any fatal TEAE	5 (2.8)	<0.1
Any TEAE leading to treatment discontinuation	8 (4.4)	<0.1
Any ISR^b^	83 (46.1)	2.8
Most common TEAEs (reported in ≥10% of participants)		
COVID-19	53 (29.4)	0.1
Headache	48 (26.7)	0.4
Nasopharyngitis	36 (20.0)	0.1
Diarrhea	32 (17.8)	0.1
Upper respiratory tract infection	32 (17.8)	0.2
Myasthenia gravis	25 (13.9)	0.1
Back pain	22 (12.2)	0.1
Anemia	19 (10.6)	0.1

^a^ER calculated as events per PYFU. ^b^The most frequently reported ISRs were erythema (57.6% of all ISR events), edema (9.0%), and swelling (7.9%). ER, event rate; ISR, injection site reaction; PYFU, participant-years of follow-up; TEAE, treatment-emergent adverse event.

**Figure 4:**
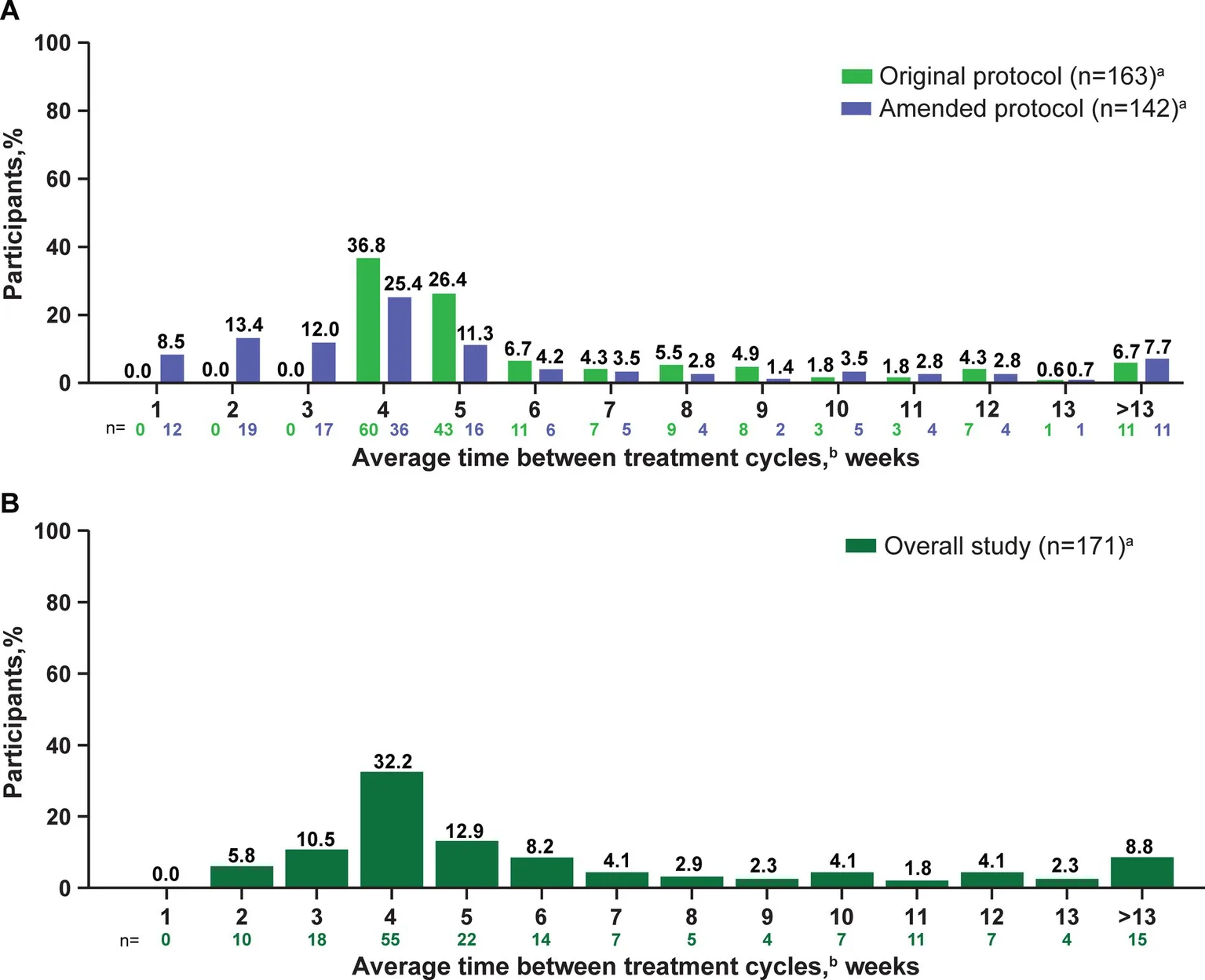
Distribution of average time between cycles among study participants **(A)** by study protocol and **(B)** during the overall study period. ^a^Participants were included in the analysis for each period if they had ≥2 treatment cycles within the relevant period. Average time between treatment cycles for each participant was calculated by dividing the total duration of all intertreatment periods by the total number of intertreatment periods.

Participants who consented to the protocol amendment (*n* = 150) received up to 20 treatment cycles (*n* = 10 participants) after the protocol amendment, with 211.8 total PYFU during amended cycles. The median (Q1, Q3) average time between treatment cycles for participants who received ≥2 treatment cycles under the original protocol (*n* = 171) was 4.8 (4.3, 7.9) weeks. Among participants who received ≥2 treatment cycles after the protocol amendment (*n* = 142), a majority (*n* = 94, 66.2%) continued to average 4 or more weeks between cycles (Figure 4A), consistent with the distribution of average time between cycles during the overall study period (Figure 4B), while 48 (33.8%) participants received more frequent treatment, with average time between cycles of 1, 2, or 3 weeks. After the protocol amendment, the median (Q1, Q3) average time between cycles decreased to 4.2 (3.0, 6.5) weeks. Safety of efgartigimod PH20 SC was evaluated in participants with more frequent dosing after the protocol amendment (average time between cycles of ≥1 to <2.5 weeks and ≥2.5 to <4 weeks) and in participants who maintained average time between cycles of ≥4 weeks. Compared with safety during pre-amendment cycles in this population, more frequent dosing did not lead to increased rates of TEAEs, severe TEAEs (grade ≥3), serious TEAEs, or ISRs (Supplementary Table 3). TEAEs leading to discontinuation of efgartigimod PH20 SC (*n* = 2, rectal cancer/metastasis to liver and Bowen’s disease) and a fatal TEAE (respiratory failure) that occurred after the protocol amendment were unrelated to efgartigimod PH20 SC treatment, as determined by investigators. In participants with AChR-Ab− gMG, rates of TEAEs, severe TEAEs, and serious TEAEs were similar to those in AChR-Ab+ participants (Supplementary Table 4).

Long-term treatment with efgartigimod PH20 SC did not lead to decreased albumin in participants, nor did treatment lead to increased total cholesterol, LDL cholesterol, or triglyceride levels (Supplementary Figures 1A–D). No clinically relevant changes in hematology, clinical chemistry, vital signs, or electrocardiogram readings were detected during ADAPT-SC+.

### 3.3 Efficacy and QoL

**Figure 5:**
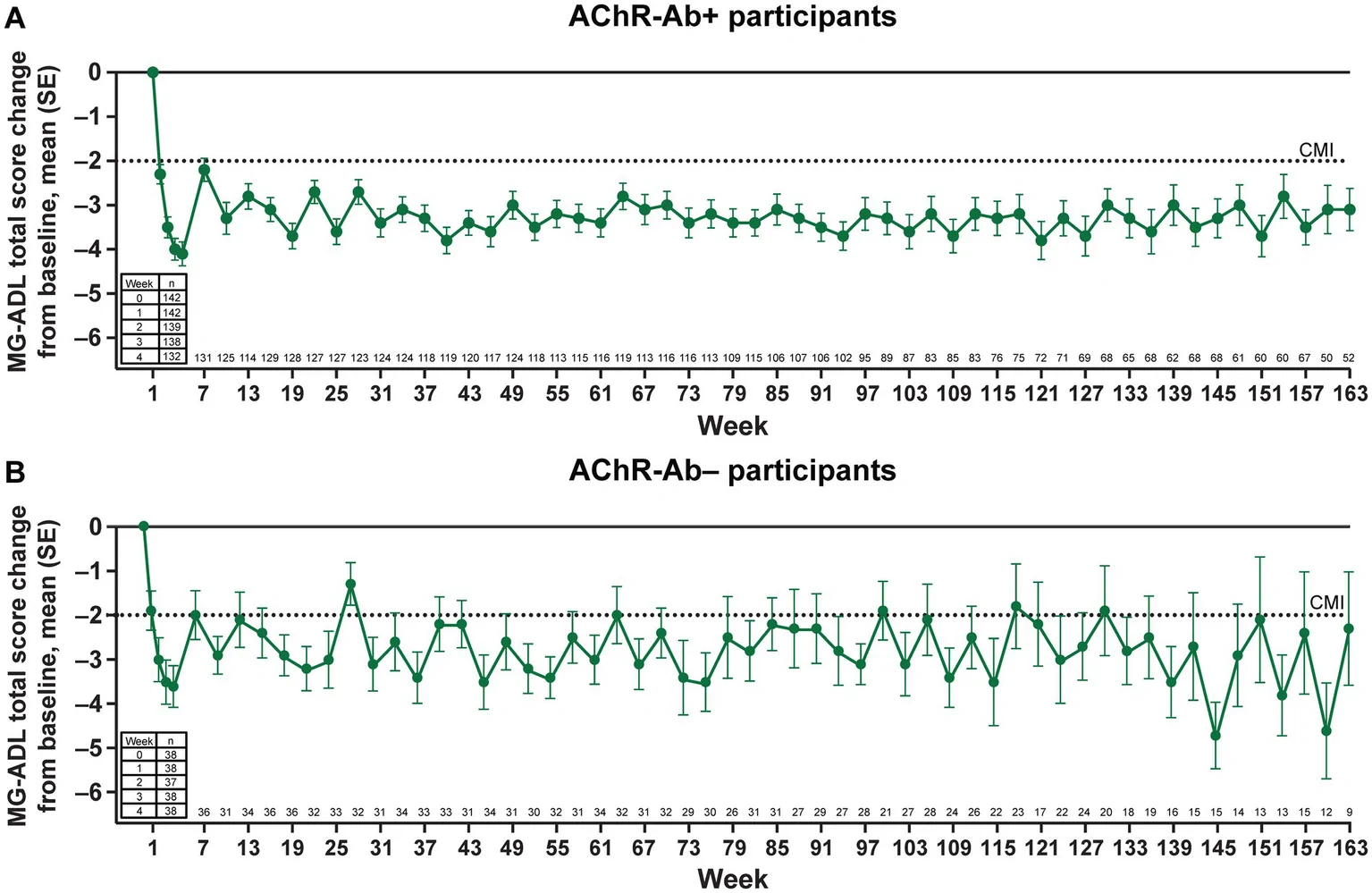
MG-ADL total score change from study baseline over time. Mean (SE) MG-ADL total score change from study baseline in participants with **(A)** AChR-Ab+ gMG or **(B)** AChR-Ab− gMG. AChR-Ab, acetylcholine receptor antibody; CMI, clinically meaningful improvement; gMG, generalized myasthenia gravis; MG-ADL, Myasthenia Gravis Activities of Daily Living.

Improvements in mean MG-ADL total scores in participants with AChR-Ab+ gMG exceeded the CMI threshold as early as 1 week after the first efgartigimod PH20 SC injection in ADAPT-SC+ and were sustained through Week 163 of the study as participants moved through treatment cycles on individualized schedules (Figure 5A). Overall, 95.1% of participants with AChR-Ab+ gMG achieved CMI at any time during the study. Improvements in participants with AChR-Ab− gMG followed a similar pattern, with the mean change in MG-ADL score exceeding the CMI threshold during most weeks in which MG-ADL was assessed (Figure 5B), and 94.7% of participants with AChR-Ab− gMG achieved CMI at any time during the study.

**Figure 6:**
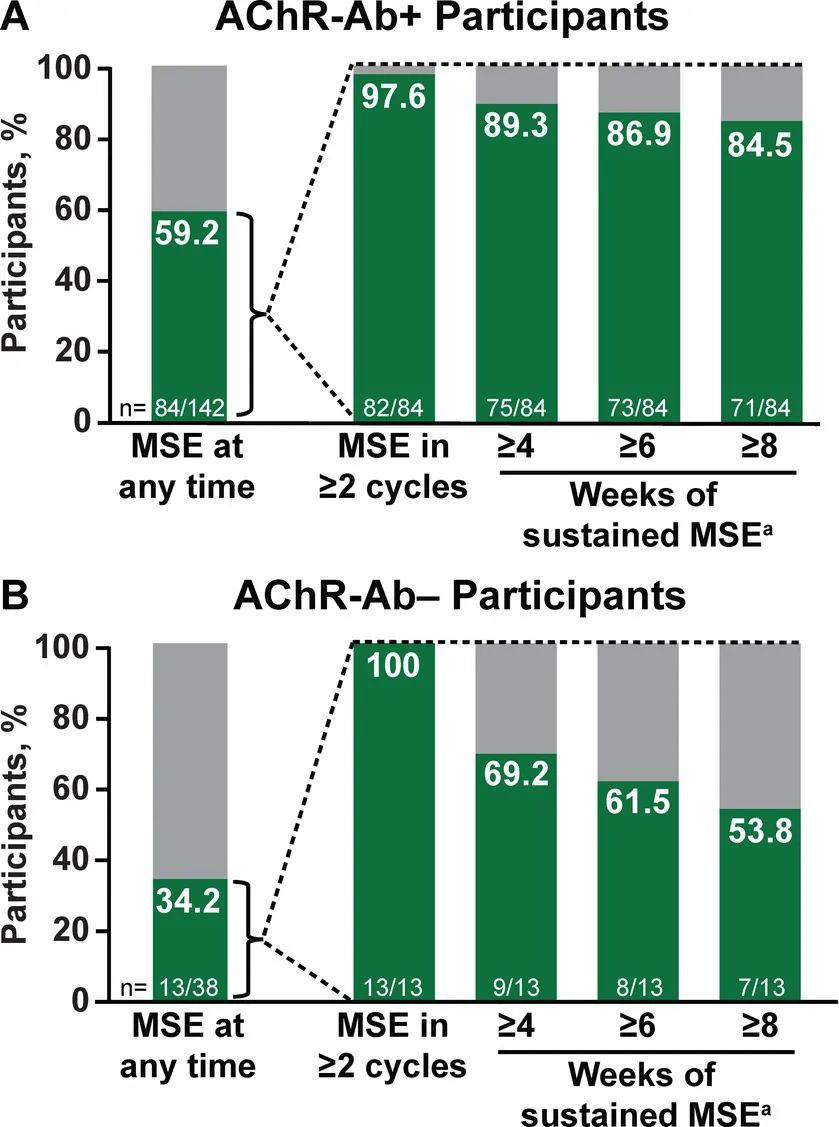
Participants achieving MSE or sustained MSE. Participants with **(A)** AChR-Ab+ gMG or **(B)** AChR-Ab− gMG who achieved MSE (MG-ADL total score, 0–1) at any time during the study, and the percentage of those who achieved MSE in ≥2 cycles or who sustained MSE in consecutive assessments across ≥4, ≥6, or ≥8 weeks. AChR-Ab, acetylcholine receptor antibody; gMG, generalized myasthenia gravis; MSE, minimal symptom expression. ^a^Percentages reflect the longest single period of sustained MSE for each participant; separate periods of MSE were not summed.

Many participants experienced and sustained MG-ADL improvements beyond the CMI threshold. Among participants with AChR-Ab+ gMG, 59.2% achieved MSE at any time during the study. Of those who achieved MSE, 97.6% experienced MSE in ≥2 treatment cycles, and most experienced sustained periods of MSE, with 89.3, 86.9, and 84.5% experiencing MSE at consecutive assessments over periods of ≥4, ≥6, and ≥8 weeks, respectively (Figure 6A). Among participants with AChR-Ab− gMG, 34.2% achieved MSE at any time during the study, all of whom achieved MSE in ≥2 treatment cycles. Most participants with AChR-Ab− gMG who experienced MSE had sustained periods of MSE, with 69.2, 61.5, and 53.8% experiencing MSE at consecutive assessments over periods of ≥4, ≥6, and ≥8 weeks, respectively (Figure 6B).

**Figure 7:**
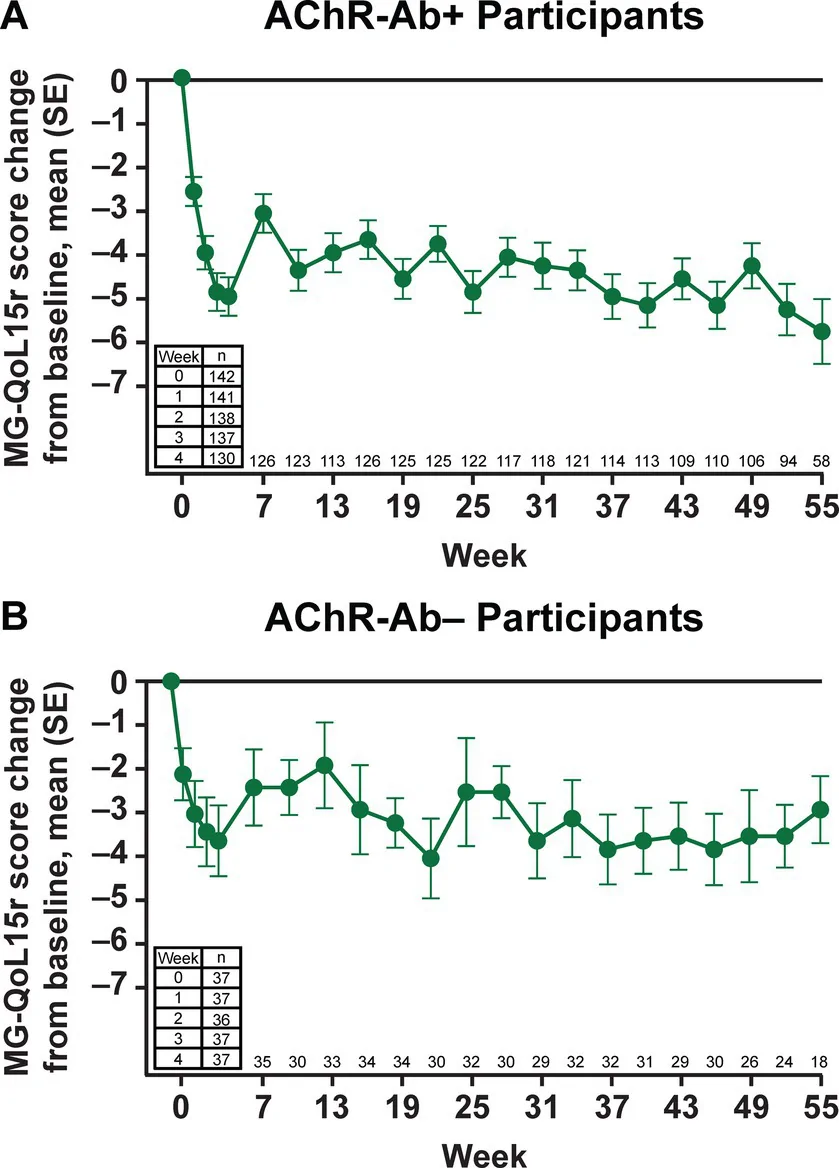
MG-QoL15r score change from study baseline over time. Mean (SE) MG-QoL15r score change from study baseline in participants with **(A)** AChR-Ab+ gMG or **(B)** AChR-Ab− gMG. AChR-Ab, acetylcholine receptor antibody; gMG, generalized myasthenia gravis; MG-QoL15r, Myasthenia Gravis Quality of Life 15-Item Questionnaire, Revised.

MG-QoL15r scores were measured during the first year of the study to assess the effect of efgartigimod PH20 SC treatment on participant QoL related to MG symptom severity. Participants with AChR-Ab+ gMG demonstrated improvement in mean MG-QoL15r score as early as Week 1 (Figure 7A). Subsequent improvements were sustained through Week 55 as participants moved through treatment cycles on individualized schedules, with the maximum mean (SE) improvement of −5.8 (0.7) points occurring at Week 55. Participants with AChR-Ab− gMG experienced a similar pattern of improvement in MG-QoL15r scores, with improvements occurring as early as Week 1 and sustained improvements observed through Week 55 (Figure 7B).

### 3.4 Pharmacodynamics

Levels of IgG and AChR-Ab were monitored during the first year of ADAPT-SC+. In the overall population, reductions in mean levels of total IgG ranged from 55.9 to 62.6% at Week 4 of each treatment cycle, relative to the mean total IgG level at the ADAPT-SC+ study baseline (Supplementary Figure 2). Among AChR-Ab+ participants, mean AChR-Ab levels followed a similar pattern, with reductions ranging from 53.8 to 59.1% at Week 4 of each treatment cycle, relative to the mean level at study baseline (Supplementary Figure 2).

## 4 Discussion

ADAPT-SC+ was an open-label extension study, with up to 3.7 years of efgartigimod PH20 SC treatment during the longest clinical trial of an FcRn blocker to date. In this final analysis, efgartigimod PH20 SC was well tolerated in a broad population of participants with gMG during a cumulative 459.4 PYFU, and clinical efficacy occurred rapidly, as early as Week 1 of the study, and was sustained throughout the study period. The inclusion of 38 participants with AChR-Ab− gMG created an opportunity to observe the safety and efficacy of efgartigimod PH20 SC in this patient population, which has been historically excluded or underrepresented in clinical trials.

Efgartigimod PH20 SC was well tolerated during ADAPT-SC+, consistent with the established safety profile of efgartigimod PH20 SC and efgartigimod IV. Among the most common TEAEs in the study were ISRs (the majority of which were injection site erythema), which is consistent with the findings in ADAPT-SC and studies of other biologic therapies, including other therapies containing PH20, administered via SC injection (23, 24, 30). Notably, all ISRs observed in ADAPT-SC+ were mild or moderate in severity, and no ISRs led to discontinuation of efgartigimod treatment. The percentage of participants experiencing ISRs decreased after the initial treatment cycle. During ADAPT-SC+, 11 participants experienced severe TEAEs of MG worsening, and 4 participants experienced myasthenic crisis. Due to the fluctuating nature of gMG symptoms, MG worsening or crisis events are often observed during long-term open-label extension studies. Rates of these events during ADAPT-SC+ were similar to those observed in other recent open-label extension studies of gMG therapies (31–34).

Infections occur at a higher rate in individuals with gMG than in the general population, which may be partially explained by the use of broad immunosuppressive therapies (35, 36). Efgartigimod treatment selectively reduces IgG levels while leaving IgG production and levels of other immunoglobulins unaffected (12, 13). Studies of efgartigimod and therapeutic plasma exchange, another gMG therapy that can reduce IgG levels, have found no correlation between decreased IgG levels and rates of infections among individuals with gMG (28, 37). During ADAPT-SC+, most infections were mild or moderate in severity, and the rate of infections and percentage of participants experiencing infections in each cycle did not increase over subsequent treatment cycles. Event rates for infections and the types and severity of infections commonly observed were consistent with previous efgartigimod clinical studies (28).

In addition to recycling IgG antibodies, FcRn also recycles albumin through binding at a site distinct from the IgG binding site, and disruption of FcRn-mediated albumin recycling can lead to reduced serum albumin levels (17, 19). Binding of some full-length monoclonal antibody FcRn inhibitors to FcRn has been shown to disrupt FcRn levels and localization, resulting in decreased albumin levels (16, 17). Low serum albumin is associated with increased risk of mortality and multiple adverse health outcomes, including cardiovascular events such as coronary artery disease and stroke, kidney function decline, and development of chronic kidney disease (17–20). Low serum albumin levels also cause dyslipidemia, which is a major contributor to development of cardiovascular disease and mortality (17, 38, 39). Binding of efgartigimod to FcRn avoids disruption of FcRn-mediated albumin recycling due to its structure as an Fc fragment (16, 17). Consistent with previous efgartigimod clinical studies, participants in ADAPT-SC+ did not experience decreased albumin levels or increases in total or LDL cholesterol upon treatment with efgartigimod PH20 SC (10, 11, 23, 40). Participants also did not experience increases in triglyceride levels. Preservation of albumin and lipid profiles during long-term treatment with efgartigimod PH20 SC provides further evidence of a favorable safety profile and supports efgartigimod as a treatment option for patients with gMG who have comorbidities, including cardiovascular comorbidities or risk factors and comorbidities with preexisting alterations in albumin levels, such as IgG-mediated nephrotic syndrome (21, 22, 41, 42).

Previous clinical trials of efgartigimod in gMG required ≥4 weeks between the final dose of a treatment cycle of 4 once-weekly doses and the initial dose of the subsequent cycle (10, 11, 23). After Year 1 of ADAPT-SC+, participants could consent to a protocol amendment permitting ≥1 week between cycles, allowing treatment to be further individualized. After the protocol amendment, about one-third of participants (n = 48) had intervals between treatment cycles that were 1, 2, or 3 weeks long on average. However, a 4-week average interval between treatment cycles was most common among participants both before and after the protocol amendment. Efgartigimod PH20 SC continued to be well tolerated in participants who consented to the amended protocol, including those with more frequent dosing after the protocol amendment. The safety of efgartigimod treatment cycles with shorter intervals between cycles is consistent with previous studies of efgartigimod for treatment of CIDP and ITP, in which weekly dosing of efgartigimod was well tolerated, with rates of TEAEs similar to those in placebo-control groups in those studies (28, 43, 44). An analysis of pooled safety data from multiple efgartigimod clinical trials across indications and formulations, which included over 2,100 participants who have been treated with efgartigimod during over 2,450 PYFU, has also demonstrated that efgartigimod is well tolerated regardless of administration route or dosing schedule (28).

Clinical efficacy and QoL improvements in participants with AChR-Ab+ gMG and participants with AChR-Ab− gMG were observed as early as Week 1 of ADAPT-SC+, and clinical improvements were sustained through Week 163 as participants received treatment cycles on individualized schedules. The majority of participants achieved CMI in MG-ADL scores. QoL was assessed during the first year of the study, and improvements were sustained during that time. Many participants achieved deeper MG-ADL improvements, with 59.2% of participants with AChR-Ab+ gMG and 34.2% of participants with AChR-Ab− gMG experiencing MSE at any time during the study. Among participants who achieved MSE at any time, most sustained MSE across consecutive assessments covering ≥8 weeks.

Observations of a consistent safety profile and clinical improvements among participants with AChR-Ab− gMG during ADAPT-SC+ add to findings of recent studies that have investigated efgartigimod IV in participants with AChR-Ab− gMG. In the Phase 3 placebo-controlled ADAPT SERON trial, participants with AChR-Ab− gMG who were treated with efgartigimod IV demonstrated significantly larger MG-ADL improvements at Week 4 of their first treatment cycle, relative to participants who received placebo. Incremental improvements were observed over subsequent cycles, and the safety profile was consistent with previous studies of efgartigimod IV (45). A recent open-label study of efgartigimod IV in participants with AChR-Ab− gMG utilized an every-2-weeks dosing approach after an initial treatment cycle and observed clinical improvements, QoL improvements, and a consistent safety profile during the study’s 23-week treatment period (46). Findings from ADAPT-SC+ add new long-term evidence suggesting that efgartigimod PH20 SC may provide a safe and efficacious treatment option for patients with AChR-Ab− gMG.

Limitations of this study include its open-label design and lack of a placebo arm. Additionally, data collection time points were limited in the study, with some data (such as IgG levels and MG-QoL15r scores) only collected during the first year of the study and other data (such as MG-ADL scores) only collected every 3 to 4 weeks during most of the study. Because of these limitations, the maximum treatment effect and full duration of the treatment effect on each measure may not have been captured. While ADAPT-SC+ included a relatively large cohort of AChR-Ab− participants, allowing insights to be drawn regarding treatment of this historically underrepresented group, MuSK-Ab and LRP4-Ab were not tested, preventing insight into the effects of efgartigimod PH20 SC in specific subgroups (ie, participants with MuSK-Ab+, LRP4-Ab+, or triple seronegative gMG) within the AChR-Ab− population.

Overall, results from ADAPT-SC+ demonstrate long-term safety, tolerability, and sustained efficacy of efgartigimod PH20 SC. Clinical benefits and QoL improvements occurred rapidly, as early as 1 week after treatment initiation, and were sustained during up to 3.7 years of treatment. Individualization of treatment cycles, even with as little as 1 week between cycles, did not alter the safety profile of efgartigimod PH20 SC. Findings from ADAPT-SC+ build on previous clinical studies with efgartigimod, offering a wider range of options to individualize treatment in a broad population of patients with gMG.

## Data Availability

argenx is committed to responsible data sharing regarding the clinical trials they fund. Included in this commitment is access to anonymized, individual, and trial-level data (analysis datasets) and other information (eg, protocols and clinical study reports), as long as the trials are not part of an ongoing or planned regulatory submission. This includes requests for clinical trial data for unlicensed products and indications. These clinical trial data can be requested by qualified researchers who engage in rigorous independent scientific research and will only be provided after review and approval of a research proposal and statistical analysis plan and execution of a data-sharing agreement. Data requests can be submitted at any time, and the data will be accessible for 12 months. Requests can be submitted to ESR@argenx.com.
