# Supplementary material for: Long-term safety, tolerability, and efficacy of subcutaneous efgartigimod PH20 in generalized myasthenia gravis: final results from a phase 3 open-label extension study (ADAPT-SC+)
Source: Front Neurol. 2026 Aug 21;17:1876344. doi: 10.3389/fneur.2026.1876344 (PMC13544598; doi:10.3389/fneur.2026.1876344)
Supplement: Supplementary file 1 [file Table_1.DOCX]

Supplementary Material

**ADAPT-SC+ Investigators**	**2**

**Supplementary Figure 1. Clinical Laboratory Measures During Treatment Cycles**	**9**

**Supplementary Figure 2. Pharmacodynamic Effect on Antibody Levels During Treatment Cycles in Year 1**	**13**

**Supplementary Table 1. Inclusion and Exclusion Criteria**	**15**

**Supplementary Table 2. Demographics and Baseline Characteristics, Amended Protocol Population**	**17**

**Supplementary Table 3. Safety, Amended Protocol Population**	**19**

**Supplementary Table 4. Safety, AChR-Ab+ and AChR-Ab**− **Populations**	**21**

**ADAPT-SC+ Investigators**

| Investigators | Study site | Country | Ethics committee(s) |
| --- | --- | --- | --- |
| Primary investigator:  Jan De Bleecker  Subinvestigator:  Annelien De Pue | AZ Sint-Lucas - Campus Sint-Lucas | Belgium | Ethische toetsingscommissie Jessa Ziekenhuis |
| Primary investigator:  Stanislav Vohanka  Subinvestigators:  Tomas Horak  Magda Horakova  Viktoria Kokosova  Michaela Sladeckova | Fakultni nemocnice Brno - Areal Bohunice | Czech Republic | Eticka komise FN Brno |
| Primary investigator:  Temur Margania  Subinvestigators:  Dali Kankava  Khatuna Sitchinava | New Hospitals | Georgia | New Hospitals |
| Primary investigator:  Roman Shakarishvili  Subinvestigators:  Elene Nebadze  Nana Kvirkvelia | Petre Sarajishvili Institute of Neurology | Georgia | Petre Sarajishvili Institute of Neurology |
| Primary investigator:  Alexander Tsiskaridze  Subinvestigators:  Lela Tavzarashvili  Diana Sanikidze  Tamar Vashadze | Pineo Medical Ecosystem | Georgia | Pineo Medical Ecosystem |
| Primary investigator:  Gvantsa Giorgadze  Subinvestigator:  Maka Mania | Aversi Clinic - Central branch | Georgia | Aversi Clinic |
| Primary investigator:  Nana Guldedava  Subinvestigator:  Nino Akiashvili | MediClub Georgia | Georgia | Medi Club Georgia LLC |
| Primary investigator:  Andreas Meisel  Subinvestigators:  Maike Krause  Hannah Pressler  Benjamin Hotter  Sarah Hoffmann  Meret Herdick  Frauke Stasheit  Carla Dusemund  Paolo Doksani | Charite Universitatsmedizin Berlin - Campus Charite Mitte (CCM) | Germany | Landesamt für Gesundheit und Soziales (LAGeSo), Geschäftsstelle der Ethik-Kommission des Landes Berlin |
| Primary investigator:  Luisa Klotz (formerly Heinz Wiendl)  Subinvestigators:  Luisa Klotz  Jan Lünemann  Catharina Korsukewitz | Universitatsklinikum Munster | Germany | Ethik-Kommission der Arztekammer Westfalen-Lippe Gartenstr |
| Primary investigator:  Csilla Rózsa  Subinvestigators:  Gabriella Varga  David Bors  Gyorgyi Szabo | Jahn Ferenc Del-pesti Korhaz es Rendelointezet | Hungary | Medical Research Council, Ethics Committee for Clinical Pharmacology  Jahn Ferenc Del-Pesti Korhaz és Rendelointezet Intezeti Kutatasetikai Bizottsag |
| Primary investigator:  Maria Judit Molnar  Subinvestigator:  Agnes Palasti | Semmelweis Egyetem - Institute of Genomics Medicine and Rare Disorders | Hungary | Medical Research Council, Ethics Committee for Clinical Pharmacology  Semmelweis Egyetem Regionalis, Intezmenyi es Tudomanyos, Kutatasetikai Bizottsag |
| Primary investigator:  Carlo Antozzi (formerly Renato Mantegazza)  Subinvestigators:  Fiammetta Vanoli  Silvia Bonanno  Lorenzo Maggi  Carlo Antozzi  Rita Frangiamore  Annamaria Gallone | Fondazione IRCCS Istituto Neurologico Carlo Besta | Italy | Comitato Etico Territoriale Lombardia 4  c/o Istituto Nazionale Tumori  Comitato Etico Della Fondazione IRCCS Istituto Neurologico “Carlo Besta” |
| Primary investigator:  Francesco Saccà  Subinvestigator:  Nunzia Cuomo | Azienda Ospedaliera Universitaria Federico II | Italy | Comitato Etico Territoriale Lombardia 4  c/o Istituto Nazionale Tumori  Comitato Etico - Università di Napoli "Federico II" c/o Dipartimento di Medicina Pubblica e della Sicurezza Sociale - Sezione di Medicina Legale |
| Primary investigator:  Stefania Morino (formerly Giovanni Antonini)  Subinvestigators:  Elena Rossini  Rocco Costanzo  Luca Leonardi  Stefania Morino  Matteo Garibaldi  Antonio Lauletta  Laura Fionda  Laura Tufano  Girolamo Alfieri | Azienda Ospedaliera Universitaria Sant'Andrea | Italy | Comitato Etico Territoriale Lombardia 4  c/o Istituto Nazionale Tumori  Comitato Etico dell'Università Sapienza  c/o Azienda Ospedaliera Sant’Andrea |
| Primary investigator:  Akiyuki Uzawa  Subinvestigators:  Yosuke Onishi  Manato Yasuda  Hiroyuki Akamine | Chiba University Hospital | Japan | Chiba University Hospital Institutional Review Board |
| Primary investigator:  Kimiaki Utsugisawa  Subinvestigator:  Yuriko Nagane | Hanamaki General Hospital | Japan | Hanamaki General Hospital Institutional Review Board |
| Primary investigator:  Genya Watanabe (formerly Yasushi Suzuki)  Subinvestigators:  Genya Watanabe  Kenichi Tsukita  Yasushi Suzuki | National Hospital Organization Sendai Medical Center | Japan | National Hospital Organization Sendai Medical Center Institutional Review Board |
| Primary investigator:  Masanori Takahashi  Subinvestigators:  Tatsusada Okuno  Tomoya Kubota | The University of Osaka Hospital | Japan | Osaka University Hospital Institutional Review Board |
| Primary investigator:  Shin Hisahara  Subinvestigators:  Kazuna Ikeda  Daisuke Yamamoto  Taro Saito  Shuichiro Suzuki  Minoru Yamada  Rei Miyanaga  Masaki Saito  Naotoshi Iwahara  Reiko Tsuda | Sapporo Medical University Hospital | Japan | Sapporo Medical University Hospital Institutional Review Board |
| Primary investigator:  Masyuki Masuda  Subinvestigators:  Nobuhiro Ido  Makiko Naito  Eri Watanabe | Tokyo Medical University Hospital | Japan | Tokyo Medical University Hospital Institutional Review Board |
| Primary investigator:  Naoya Minami  Subinvestigators:  Itaru Amino  Masaaki Niino  Yusei Miyazaki  Sachiko Akimoto  Ryoji Naganuma | National Hospital Organization Hokkaido Medical Center | Japan | National Hospital Organization Hokkaido Medical Center Institutional Review Board |
| Primary investigator:  Takehisa Hirayama (formerly Kiyokazu Kawabe)  Subinvestigators:  Takehisa Hirayama  Kiyokazu Kawabe  Masaru Yanagihashi | Toho University Omori Medical Center | Japan | Toho University Omori Medical Center Institutional Review Board |
| Primary investigator:  Jan JGM Verschuuren  Subinvestigators:  Martijn Tannemaat  Kevin Keene  Annabel Ruiter  Linda Remijn-Nelissen  Yvonne Campman | Leids Universitair Medisch Centrum | Netherlands | MREC Leiden The Hague Delft |
| Primary investigator:  Malgorzata Bilinska  Subinvestigator:  Marek Halas | Uniwersyteckie Centrum Kliniczne w Gdansku | Poland | Komisja Bioetyczna ds. Badan Naukowych |
| Primary investigator:  Andrzej Szczudlik  Subinvestigators:  Grazyna Zwolinska  Dorota Zawislak | Krakowska Akademia Neurologii Sp. z o.o. | Poland | Ethics Committee at the Regional Chamber of Physicians in Krakow |
| Primary investigator:  Marek Smilowski  Subinvestigators:  Lech Bronislaw Szczechowski  Piotr Zych  Anna Starostka-Tatar | Wielospecjalistyczna Poradnia Lekarska Synapsis | Poland | Komisja Bioetyczna przy Slaskiej Izbie Lekarskiej |
| Primary investigator:  Urszula Chyrchel-Paszkiewicz  Subinvestigator:  Magdalena Ogorek | CLINIREM Sp z o.o. - Lublinie | Poland | Komisja Bioetyczna przy Lubelskiej Izbie Lekarskiej |
| Primary investigator:  Anna Kostera-Pruszczyk  Subinvestigators:  Piotr Szczudlik  Ewa Sobieszczuk  Aleksandra Golenia | Centralny Szpital Kliniczny - Uniwersyteckie Centrum Kliniczne WUM | Poland | Komisja Bioetyczna przy Warszawskim Uniwersytecie Medycznym |
| Primary investigator:  Agnieszka Kulaga (formerly Krzysztof Banaszkiewicz)  Subinvestigators:  Michal Blaz  Ewa Majcherczyk  Krystian Majkowski  Michal Michalski  Kacper Szewczy | Krakowski Szpital Specjalistyczny im sw. Jana Pawla II | Poland | Ethics Committee at the Regional Chamber of Physicians in Krakow |
| Primary investigator:  Denis Korobko (formerly Nadezhda Malkova)  Subinvestigators:  Ilona Vergunova  Ekaterina Tretyakova (Ekaterina Bulatova)  Anna Prokaeva | Novosibirsk State Regional Hospital | Russia | Ethics Committee at the GBUZ (State Budgetary Healthcare Institution) of Novosibirsk Region State, Novosibirsk Regional Clinical Hospital |
| Primary investigator:  Fatima Stuchevskaya  Subinvestigators:  Anna Abramova  Mariia Vasileva | City Multi-field Hospital #2 | Russia | Ethics Committee at Medical Center Reavita Med SPb LLC |
| Primary investigator:  Raúl Juntas Morales (formerly Maria Salvado-Figueras)  Subinvestigators:  Raul Juntas Morales  Daniel Sanchez-Tejerina San Jose  Arnau Llaurado  Veronica Lopez  Javier Joaquín Sotoca | Hospital Universitario Vall d'Hebron | Spain | CEIm Hospital Clinico San Carlos |
| Primary investigator:  Elena Cortes Vicente  Subinvestigators:  Rodrigo Alvarez Velasco  David Reyes Leiva  Ana Vesperinas | Hospital de la Santa Creu i Sant Pau | Spain | CEIm Hospital Clinico San Carlos |
| Primary investigator:  Teresa Sevilla Mantecón  Subinvestigators:  Marina Frasquet Carrera  Juan Sebastián Aller  Francisco Javier Cabello  Jesus Jimenez  Javier Caballero  Rafael Sivera | Hospital Universitari i Politecnic La Fe de Valencia | Spain | CEIm Hospital Clinico San Carlos |
| Primary investigator:  James F Howard, Jr  Subinvestigator:  Rebecca Traub | UNC Health - UNC Hospitals | USA | Advarra Institutional Review Board |
| Primary investigator:  Tuan Vu  Subinvestigators:  Jerrica Farias  Niraja Sathyanarayanan Suresh | University of South Florida (USF) Health - Morsani Center for Advanced Healthcare | USA | Advarra Institutional Review Board |
| Primary investigator:  Tulio Bertorini  Subinvestigators:  Cindy Benzel  Amanda Huffman  Yaohui Chai | Neurology Clinic PC | USA | Advarra Institutional Review Board |
| Primary investigator:  Ratna Bhavaraju-Sanka  Subinvestigators:  Carlayne Jackson  Lorelei Salazar | UT Health - University of Texas Health Science Center at San Antonio | USA | Advarra Institutional Review Board |
| Primary investigator:  Mamatha Pasnoor  Subinvestigators:  Jeffrey Statland  Constantine Farmakidis  Omar Jawdat  Duaa Jabari  Mazen Dimachkie  Mathew Varon | Kansas University Medical Center - Kansas City | USA | Advarra Institutional Review Board |
| Primary investigator:  Yuebing Li  Subinvestigator:  Benjamin Claytor | Cleveland Clinic - Main Campus | USA | Cleveland Clinic Institutional Review Board |
| Primary investigator:  Srikanth Muppidi  Subinvestigators:  Yuen Tat So  Neelam Goyal | Stanford Health Care - Stanford Neuroscience Health Center | USA | Stanford University Research Compliance Office |
| Primary investigator:  Gregory Sahagian  Subinvestigators:  John Heinen  Kinjal Madhav  Tara Quesnell  Benjamin Frishberg  Guadalupe Sanchez  April Tenorio  Estela Soto  Angela Sutherland  Yiyun Pan | The Neurology Center of Southern California - Carslbad Office | USA | Advarra Institutional Review Board |
| Primary investigator:  Yessar Hussain  Subinvestigator:  Casey Kafena | National Neuromuscular Research Institute | USA | Advarra Institutional Review Board |
| Primary investigator:  Vern Juel (formerly Jeffrey Guptil)  Subinvestigators:  Natalia Laura Gonzalez  Moboluwade D. Abe-Lathan  Ellen Tuck  Shruti M. Raja | Duke University School of Medicine - Duke Early Phase Clinical Research Unit | USA | Duke University Health System Institutional Review Board for Clinical Investigations |
| Primary investigator:  Brian Costell  Subinvestigators:  John Nangle  Chi Dargelis | Neurology Offices of South Florida | USA | Advarra Institutional Review Board |
| Primary investigator:  Luisa Rojas Estupinan  Subinvestigators:  Anna Mattle  Katelyn McCormack  Wendy Callen  Allison Embrosky  Tanya Geist  Michelle Rainka  Traci Aladeen  Bennett H. Myers | Dent Neurologic Institute - Amherst | USA | Advarra Institutional Review Board |
| Primary investigator:  George Li  Subinvestigators:  David Klein  Jana Gray  Victoria Georgoulis | Medsol Clinical Research Center Inc | USA | Advarra Institutional Review Board |

**Supplementary Figure 1A**

**Supplementary Figure 1.** Clinical Laboratory Measures During Treatment Cycles. For each cycle, measures at Weeks 0 (baseline), 4, and 7 are shown. (**A**) Concentration of albumin. (**B**) Concentration of total cholesterol. (**C**) Concentration of LDL cholesterol. (**D**) Concentration of triglycerides. LDL, low-density lipoprotein; LLN, lower limit of normal; ULN, upper limit of normal.

**Supplementary Figure 1B**

**Supplementary Figure 1C**

**Supplementary Figure 1D**

**Supplementary Figure 2A**

**Supplementary Figure 2.** Pharmacodynamic Effect on Antibody Levels During Treatment Cycles in Year 1. For each cycle, measures at Weeks 0 (baseline), 4, and 7 are shown. (**A**) Levels of total IgG among participants in the overall population. (**B**) Levels of AChR-Ab among participants with AChR-Ab+ gMG. ^a^Samples were excluded if taken after an IgG-related MG therapy or plasmapheresis procedure was initiated during a cycle. AChR-Ab, acetylcholine receptor antibody; gMG, generalized myasthenia gravis; IgG, immunoglobulin G; MG, myasthenia gravis.

**Supplementary Figure 2B**

**Supplementary Table 1. Inclusion and Exclusion Criteria**

| Inclusion criteria | Participants were eligible to be included in ADAPT-SC+ only if all of the following criteria were met:   1. Participants were capable of giving signed informed consent, which includes compliance with the requirements and restrictions listed in the ICF and in the study protocol 2. Previously participated in antecedent studies ADAPT-SC or ADAPT+ and were eligible for rollover as defined by:    1. For ADAPT-SC       1. Completed the study and performed the end-of-study visit, or       2. Were discontinued from study treatment for reasons other than pregnancy or a life-threatening SAE. Receiving rescue therapy was not exclusionary unless given in a response to a life-threatening situation    2. For ADAPT+       1. Performed the end of Part A, or       2. Started Part B, received the previous dose of efgartigimod IV at least 30 days prior to entry into this study, completed at least 1 year of ADAPT+, and performed the early discontinuation visit in ADAPT+       3. Did not have 3 consecutive treatment failures in ADAPT+ Part A, even if the participant received rescue therapy (unless rescue therapy was given in response to a life-threatening situation). Treatment failure was defined as the absence of a decrease of at least 2 points in total MG-ADL score compared to the subsequent treatment cycle baseline in at least 50% of the assessments       4. Are still receiving concomitant gMG medication. Participants who stopped taking any concomitant medication for gMG were not eligible for rollover 3. Women of childbearing potential needed to have a negative urine pregnancy test at baseline before efgartigimod could be administered and needed to use a highly effective or acceptable method of contraception until ≥90 days after the last dose of efgartigimod PH20 SC. Contraceptive use by male and female participants needed to follow local regulations regarding the methods of contraception for those participating in clinical studies |
| --- | --- |
| Exclusion criteria | Participants were excluded from ADAPT-SC+ if any of the following criteria applied:   1. The participant was discontinued early from studies ADAPT-SC or ADAPT+, unless the reason for discontinuation from study ADAPT+ was to roll over into ADAPT-SC+    1. Participants who, in the investigator’s judgment, were not benefiting from efgartigimod IV in ADAPT+ Part B were not eligible for rollover into ADAPT-SC+ 2. Participant was pregnant or lactating, or intended to become pregnant during the study or within 90 days after the last dose of efgartigimod PH20 SC 3. Participant had any of the following medical conditions:    1. Clinically significant uncontrolled chronic bacterial, viral, or fungal infection at rollover    2. Any other known autoimmune disease that, in the opinion of the investigator, would interfere with accurate assessment of clinical symptoms of myasthenia gravis or put the participant at undue risk    3. History of malignancy unless deemed cured by adequate treatment with no evidence of reoccurrence for ≥3 years before the first administration of efgartigimod PH20 SC. Participants with the following cancers can be included at any time:       1. Adequately treated basal cell or squamous cell skin cancer       2. Carcinoma in situ of the cervix       3. Carcinoma in situ of the breast       4. Incidental histological findings of prostate cancer (TNM classification of malignant tumors stage T1a or T1b)    4. Clinical evidence of other significant serious diseases, or the participant had a recent major surgery, or the participant had any other condition that, in the opinion of the investigator, could confound the results of the study or put the participant at undue risk 4. Participant received a live-attenuated vaccine within 28 days prior to study entry or plans to receive a live-attenuated vaccine during the study 5. Participant had a known hypersensitivity reaction to efgartigimod, rHuPH20, or any of its excipients |

ICF, informed consent form; IV, intravenous; gMG, generalized myasthenia gravis; MG-ADL, Myasthenia Gravis Activities of Daily Living; rHuPH20, recombinant human hyaluronidase PH20; SAE, serious adverse event; SC, subcutaneous; TNM, tumor, nodes, metastasis.

**Supplementary Table 2. Demographics and Baseline Characteristics, Amended Protocol Population**

|  | **Overall (n=150)** |
| --- | --- |
| Age (y) |  |
| Mean (SD) | 50.2 (15.3) |
| Median (min, max) | 50.0 (19, 84) |
| Sex, n (%) |  |
| Female | 103 (68.7) |
| Male | 47 (31.3) |
| Race, n (%) |  |
| Asian (Japanese) | 15 (10.0) |
| Black or African American | 2 (1.3) |
| Multiple | 1 (0.7) |
| White | 132 (88.0) |
| Weight (kg) |  |
| Mean (SD) | 78.1 (20.2) |
| Median (min, max) | 76.2 (42.0, 148.8) |
| Time since gMG diagnosis (y) |  |
| Mean (SD) | 9.5 (8.3) |
| Median (min, max) | 7.2 (0.4, 48.1) |
| Previous thymectomy, n (%) |  |
| Yes | 58 (38.7) |
| MGFA class at screening, n (%) |  |
| Class II | 61 (40.7) |
| Class III | 82 (54.7) |
| Class IV | 7 (4.7) |
| MG-ADL total score at baseline |  |
| Mean (SD) | 7.7 (3.5) |
| Median (min, max) | 7.0 (0, 20) |
| MG-QoL15r total score at baseline |  |
| Mean (SD) | 13.0 (6.9) |
| Median (min, max) | 12.0 (0, 30) |
| AChR-Ab status, n (%) |  |
| AChR-Ab+ | 118 (78.7) |
| AChR-Ab− | 32 (21.3) |
| Concomitant^a^ MG therapy, n (%) |  |
| Any steroid | 106 (70.7) |
| Any NSIST | 77 (51.3) |
| Any AChEI | 123 (82.0) |
| Steroid + NSIST | 57 (38.0) |
| AChEI only | 23 (15.3) |

AChEI, acetylcholinesterase inhibitor; AChR-Ab, acetylcholine receptor antibody; gMG, generalized myasthenia gravis; MG, myasthenia gravis; MG-ADL, Myasthenia Gravis Activities of Daily Living; MGFA, Myasthenia Gravis Foundation of America; MG-QoL15r, Myasthenia Gravis Quality of Life 15-Item Questionnaire, Revised; NSIST, nonsteroidal immunosuppressive therapy.

**Supplementary Table 3. Safety, Amended Protocol Population**

|  | **During pre-amendment cycles  (n=146, 220.7 PYFU)** | | **Post-amendment cycles** | | | | | | | |
| --- | --- | --- | --- | --- | --- | --- | --- | --- | --- | --- |
|  |  |  | **Lowest exposure,  average IP ≥4 weeks (n=79; 118.9 PYFU)** | | **Intermediate exposure, average IP ≥2.5 to <4 weeks (n=39; 46.0 PYFU)** | | **Highest exposure,  average IP ≥1 to <2.5 weeks (n=32; 46.9 PYFU)** | | | |
|  | **n (%)** | **ER^a^** | **n (%)** | **ER^a^** | **n (%)** | **ER^a^** | **n (%)** | | **ER^a^** | |
| Any TEAE | 128 (87.7) | 8.8 | 64 (81.0) | 6.0 | 33 (84.6) | 7.1 | 22 (68.8) | | 2.7 | |
| Any TEAE grade ≥3 | 33 (22.6) | 0.3 | 17 (21.5) | 0.3 | 9 (23.1) | 0.3 | 4 (12.5) | | 0.1 | |
| Any serious TEAE | 25 (17.1) | 0.2 | 13 (16.5) | 0.2 | 8 (20.5) | 0.2 | 3 (9.4) | | 0.1 | |
| Any fatal TEAE | 0 | − | 1 (1.3) | <0.1 | 0 | − | 0 | | − | |
| Any TEAE leading to treatment discontinuation | 0 | − | 1 (1.3) | <0.1 | 1 (2.6) | <0.1 | 0 | | − | |
| Any ISR^b^ | 70 (47.9) | 3.2 | 16 (20.3) | 3.0 | 5 (12.8) | 2.7 | 2 (6.3) | | 0.1 | |
| Most common TEAEs (reported in ≥10% of overall population participants) | | | | | | | |  | |  |
| COVID-19 | 40 (27.4) | 0.2 | 7 (8.9) | 0.1 | 4 (10.3) | 0.1 | 2 (6.3) | | 0.1 | |
| Headache | 34 (23.3) | 0.6 | 13 (16.5) | 0.3 | 4 (10.3) | 0.5 | 4 (12.5) | | 0.1 | |
| Nasopharyngitis | 29 (19.9) | 0.2 | 7 (8.9) | 0.1 | 4 (10.3) | 0.1 | 1 (3.1) | | 0.1 | |
| Diarrhea | 20 (13.7) | 0.1 | 3 (3.8) | <0.1 | 5 (12.8) | 0.1 | 1 (3.1) | | 0.1 | |
| Upper respiratory tract infection | 22 (15.1) | 0.2 | 9 (11.4) | 0.1 | 8 (20.5) | 0.2 | 4 (12.5) | | 0.2 | |
| Myasthenia gravis worsening | 14 (9.6) | 0.1 | 1 (1.3) | <0.1 | 3 (7.7) | 0.1 | 4 (12.5) | | <0.1 | |
| Back pain | 12 (8.2) | 0.1 | 4 (5.1) | <0.1 | 3 (7.7) | 0.1 | 1 (3.1) | | 0.1 | |
| Anemia | 13 (8.9) | 0.1 | 3 (3.8) | 0.1 | 2 (5.1) | 0.1 | 3 (9.4) | | 0.1 | |

^a^ER calculated as events per PYFU. ^b^The most frequently reported ISRs in the overall population were erythema, edema, and swelling. ER, event rate; IP, intertreatment period; ISR, injection site reaction; PYFU, participant years of follow-up; TEAE, treatment-emergent adverse event.

**Supplementary Table 4. Safety, AChR-Ab+ and AChR-Ab− Populations**

|  | **AChR-Ab+ population  (n=142, 362.1 PYFU)** | | **AChR-Ab− population  (n=38, 97.4 PYFU)** | |
| --- | --- | --- | --- | --- |
|  | **n (%)** | **ER^a^** | **n (%)** | **ER^a^** |
| Any TEAE | 132 (93.0) | 6.8 | 37 (97.4) | 9.0 |
| Any TEAE grade ≥3 | 50 (35.2) | 0.3 | 12 (31.6) | 0.4 |
| Any serious TEAE | 44 (31.0) | 0.2 | 11 (28.9) | 0.2 |
| Any fatal TEAE | 5 (3.5) | <0.1 | 0 | 0 |
| Any TEAE leading to treatment discontinuation | 6 (4.2) | <0.1 | 2 (5.3) | <0.1 |
| Any ISR^b^ | 61 (43.0) | 2.5 | 22 (57.9) | 3.6 |
| Most common TEAEs (reported in ≥10% of overall population participants) | | | |  |
| COVID-19 | 39 (27.5) | 0.1 | 14 (36.8) | 0.2 |
| Headache | 38 (26.8) | 0.5 | 10 (26.3) | 0.2 |
| Nasopharyngitis | 27 (19.0) | 0.1 | 9 (23.7) | 0.1 |
| Upper respiratory tract infection | 23 (16.2) | 0.2 | 9 (23.7) | 0.2 |
| Diarrhea | 23 (16.2) | 0.1 | 9 (23.7) | 0.2 |
| Myasthenia gravis worsening | 18 (12.7) | 0.1 | 7 (18.4) | 0.1 |
| Back pain | 13 (9.2) | <0.1 | 9 (23.7) | 0.1 |
| Anemia | 15 (10.6) | 0.1 | 4 (10.5) | 0.1 |

^a^ER calculated as events per PYFU. ^b^The most frequently reported ISRs in the overall population were erythema, edema, and swelling. AChR-Ab, acetylcholine receptor antibody; ER, event rate; ISR, injection site reaction; PYFU, participant years of follow-up; TEAE, treatment-emergent adverse event.
